# Supplementary material for: Resolving the full spectrum of human genome variation using Linked-Reads
Source: Genome Res. 2019 Apr;29(4):635–45. doi: 10.1101/gr.234443.118 (PMC6442396; doi:10.1101/gr.234443.118)
Supplement: Supplemental Material [file supp_gr.234443.118_Supplemental_longranger-2.2.zip › longranger-2.2.tar.bz2!/lr2.2-public/lib/stan/cmdstan/stan/doc/api/html/term__grammar__def_8hpp.html]

Stan: src/stan/gm/grammars/term\_grammar\_def.hpp File Reference


|  |  |
| --- | --- |
|  | Stan  2.5.0  probability, sampling & optimization |


- Main Page
- Related Pages
- Namespaces
- Classes
- Files

- File List
- File Members

All Classes Namespaces Files Functions Variables Typedefs Enumerations Enumerator Friends Macros Pages

- src
- stan
- gm
- grammars

Classes |
Namespaces |
Functions |
Variables

term\_grammar\_def.hpp File Reference

`#include <cstddef>`  
`#include <iomanip>`  
`#include <iostream>`  
`#include <istream>`  
`#include <map>`  
`#include <set>`  
`#include <sstream>`  
`#include <string>`  
`#include <utility>`  
`#include <vector>`  
`#include <stdexcept>`  
`#include <boost/spirit/include/qi.hpp>`  
`#include <boost/spirit/include/phoenix_core.hpp>`  
`#include <boost/spirit/include/phoenix_function.hpp>`  
`#include <boost/spirit/include/phoenix_fusion.hpp>`  
`#include <boost/spirit/include/phoenix_object.hpp>`  
`#include <boost/spirit/include/phoenix_operator.hpp>`  
`#include <boost/spirit/include/phoenix_stl.hpp>`  
`#include <boost/lexical_cast.hpp>`  
`#include <boost/fusion/include/adapt_struct.hpp>`  
`#include <boost/fusion/include/std_pair.hpp>`  
`#include <boost/config/warning_disable.hpp>`  
`#include <boost/spirit/include/qi_numeric.hpp>`  
`#include <boost/spirit/include/classic_position_iterator.hpp>`  
`#include <boost/spirit/include/support_multi_pass.hpp>`  
`#include <boost/tuple/tuple.hpp>`  
`#include <boost/variant/apply_visitor.hpp>`  
`#include <boost/variant/recursive_variant.hpp>`  
`#include <stan/gm/ast.hpp>`  
`#include <stan/gm/grammars/whitespace_grammar.hpp>`  
`#include <stan/gm/grammars/term_grammar.hpp>`  
`#include <stan/gm/grammars/expression_grammar.hpp>`

Go to the source code of this file.

|  |  |
| --- | --- |
| Classes | |
| struct | stan::gm::validate\_integrate\_ode |
|  | |
| struct | stan::gm::validate\_integrate\_ode::result< T1, T2, T3, T4 > |
|  | |
| struct | stan::gm::set\_fun\_type |
|  | |
| struct | stan::gm::set\_fun\_type::result< T1, T2 > |
|  | |
| struct | stan::gm::set\_fun\_type\_named |
|  | |
| struct | stan::gm::set\_fun\_type\_named::result< T1, T2, T3, T4, T5 > |
|  | |
| struct | stan::gm::exponentiation\_expr |
|  | |
| struct | stan::gm::exponentiation\_expr::result< T1, T2, T3, T4, T5 > |
|  | |
| struct | stan::gm::multiplication\_expr |
|  | |
| struct | stan::gm::multiplication\_expr::result< T1, T2, T3 > |
|  | |
| struct | stan::gm::division\_expr |
|  | |
| struct | stan::gm::division\_expr::result< T1, T2, T3 > |
|  | |
| struct | stan::gm::modulus\_expr |
|  | |
| struct | stan::gm::modulus\_expr::result< T1, T2, T3, T4 > |
|  | |
| struct | stan::gm::left\_division\_expr |
|  | |
| struct | stan::gm::left\_division\_expr::result< T1, T2, T3 > |
|  | |
| struct | stan::gm::elt\_multiplication\_expr |
|  | |
| struct | stan::gm::elt\_multiplication\_expr::result< T1, T2, T3 > |
|  | |
| struct | stan::gm::elt\_division\_expr |
|  | |
| struct | stan::gm::elt\_division\_expr::result< T1, T2, T3 > |
|  | |
| struct | stan::gm::negate\_expr |
|  | |
| struct | stan::gm::negate\_expr::result< T1, T2, T3, T4 > |
|  | |
| struct | stan::gm::logical\_negate\_expr |
|  | |
| struct | stan::gm::logical\_negate\_expr::result< T1, T2, T3 > |
|  | |
| struct | stan::gm::transpose\_expr |
|  | |
| struct | stan::gm::transpose\_expr::result< T1, T2 > |
|  | |
| struct | stan::gm::add\_expression\_dimss |
|  | |
| struct | stan::gm::add\_expression\_dimss::result< T1, T2, T3, T4 > |
|  | |
| struct | stan::gm::set\_var\_type |
|  | |
| struct | stan::gm::set\_var\_type::result< T1, T2, T3, T4 > |
|  | |
| struct | stan::gm::validate\_int\_expr3 |
|  | |
| struct | stan::gm::validate\_int\_expr3::result< T1, T2 > |
|  | |

|  |  |
| --- | --- |
| Namespaces | |
|  | stan |
|  | Probability, optimization and sampling library. |
|  | |
|  | stan::gm |
|  | Directed graphical model compiler. |
|  | |

|  |  |
| --- | --- |
| Functions | |
|  | BOOST\_FUSION\_ADAPT\_STRUCT (stan::gm::index\_op,(stan::gm::expression, expr\_)(std::vector< std::vector< stan::gm::expression > >, dimss\_)) |
|  | |
|  | BOOST\_FUSION\_ADAPT\_STRUCT (stan::gm::integrate\_ode,(std::string, system\_function\_name\_)(stan::gm::expression, y0\_)(stan::gm::expression, t0\_)(stan::gm::expression, ts\_)(stan::gm::expression, theta\_)(stan::gm::expression, x\_)(stan::gm::expression, x\_int\_)) |
|  | |
|  | BOOST\_FUSION\_ADAPT\_STRUCT (stan::gm::fun,(std::string, name\_)(std::vector< stan::gm::expression >, args\_)) |
|  | |
|  | BOOST\_FUSION\_ADAPT\_STRUCT (stan::gm::int\_literal,(int, val\_)(stan::gm::expr\_type, type\_)) |
|  | |
|  | BOOST\_FUSION\_ADAPT\_STRUCT (stan::gm::double\_literal,(double, val\_)(stan::gm::expr\_type, type\_)) |
|  | |
| void | stan::gm::generate\_expression (const expression &e, std::ostream &o) |
|  | |

|  |  |
| --- | --- |
| Variables | |
| boost::phoenix::function  < validate\_integrate\_ode > | stan::gm::validate\_integrate\_ode\_f |
|  | |
| boost::phoenix::function  < set\_fun\_type > | stan::gm::set\_fun\_type\_f |
|  | |
| boost::phoenix::function  < set\_fun\_type\_named > | stan::gm::set\_fun\_type\_named\_f |
|  | |
| boost::phoenix::function  < exponentiation\_expr > | stan::gm::exponentiation\_f |
|  | |
| boost::phoenix::function  < multiplication\_expr > | stan::gm::multiplication\_f |
|  | |
| boost::phoenix::function  < division\_expr > | stan::gm::division\_f |
|  | |
| boost::phoenix::function  < modulus\_expr > | stan::gm::modulus\_f |
|  | |
| boost::phoenix::function  < left\_division\_expr > | stan::gm::left\_division\_f |
|  | |
| boost::phoenix::function  < elt\_multiplication\_expr > | stan::gm::elt\_multiplication\_f |
|  | |
| boost::phoenix::function  < elt\_division\_expr > | stan::gm::elt\_division\_f |
|  | |
| boost::phoenix::function  < negate\_expr > | stan::gm::negate\_expr\_f |
|  | |
| boost::phoenix::function  < logical\_negate\_expr > | stan::gm::logical\_negate\_expr\_f |
|  | |
| boost::phoenix::function  < transpose\_expr > | stan::gm::transpose\_f |
|  | |
| boost::phoenix::function  < add\_expression\_dimss > | stan::gm::add\_expression\_dimss\_f |
|  | |
| boost::phoenix::function  < set\_var\_type > | stan::gm::set\_var\_type\_f |
|  | |
| boost::phoenix::function  < validate\_int\_expr3 > | stan::gm::validate\_int\_expr3\_f |
|  | |

## Function Documentation

|  |  |  |  |
| --- | --- | --- | --- |
| BOOST\_FUSION\_ADAPT\_STRUCT | ( | stan::gm::index\_op | , |
|  |  | (stan::gm::expression, expr\_)(std::vector< std::vector< stan::gm::expression > >, dimss\_) |  |
|  | ) |  |  |

|  |  |  |  |
| --- | --- | --- | --- |
| BOOST\_FUSION\_ADAPT\_STRUCT | ( | stan::gm::integrate\_ode | , |
|  |  | (std::string, system\_function\_name\_)(stan::gm::expression, y0\_)(stan::gm::expression, t0\_)(stan::gm::expression, ts\_)(stan::gm::expression, theta\_)(stan::gm::expression, x\_)(stan::gm::expression, x\_int\_) |  |
|  | ) |  |  |

|  |  |  |  |
| --- | --- | --- | --- |
| BOOST\_FUSION\_ADAPT\_STRUCT | ( | stan::gm::fun | , |
|  |  | (std::string, name\_)(std::vector< stan::gm::expression >, args\_) |  |
|  | ) |  |  |

|  |  |  |  |
| --- | --- | --- | --- |
| BOOST\_FUSION\_ADAPT\_STRUCT | ( | stan::gm::int\_literal | , |
|  |  | (int, val\_)(stan::gm::expr\_type, type\_) |  |
|  | ) |  |  |

|  |  |  |  |
| --- | --- | --- | --- |
| BOOST\_FUSION\_ADAPT\_STRUCT | ( | stan::gm::double\_literal | , |
|  |  | (double, val\_)(stan::gm::expr\_type, type\_) |  |
|  | ) |  |  |


---

[ Stan Home Page ]

*© 2011–2014,
Stan Development Team.*
